# Dependency-based Siamese long short-term memory network for learning sentence representations

**DOI:** 10.1371/journal.pone.0193919

**Published:** 2018-03-07

**Authors:** Wenhao Zhu, Tengjun Yao, Jianyue Ni, Baogang Wei, Zhiguo Lu

**Affiliations:** 1 School of Computer Engineering and Science, Shanghai University, Shanghai, China; 2 College of Computer Science and Technology, Zhejiang University, Zhejiang, China; 3 Library of Shanghai University, Shanghai University, Shanghai, China; Hangzhou Normal University, CHINA

## Abstract

Textual representations play an important role in the field of natural language processing (NLP). The efficiency of NLP tasks, such as text comprehension and information extraction, can be significantly improved with proper textual representations. As neural networks are gradually applied to learn the representation of words and phrases, fairly efficient models of learning short text representations have been developed, such as the continuous bag of words (CBOW) and skip-gram models, and they have been extensively employed in a variety of NLP tasks. Because of the complex structure generated by the longer text lengths, such as sentences, algorithms appropriate for learning short textual representations are not applicable for learning long textual representations. One method of learning long textual representations is the Long Short-Term Memory (LSTM) network, which is suitable for processing sequences. However, the standard LSTM does not adequately address the primary sentence structure (subject, predicate and object), which is an important factor for producing appropriate sentence representations. To resolve this issue, this paper proposes the dependency-based LSTM model (D-LSTM). The D-LSTM divides a sentence representation into two parts: a basic component and a supporting component. The D-LSTM uses a pre-trained dependency parser to obtain the primary sentence information and generate supporting components, and it also uses a standard LSTM model to generate the basic sentence components. A weight factor that can adjust the ratio of the basic and supporting components in a sentence is introduced to generate the sentence representation. Compared with the representation learned by the standard LSTM, the sentence representation learned by the D-LSTM contains a greater amount of useful information. The experimental results show that the D-LSTM is superior to the standard LSTM for sentences involving compositional knowledge (SICK) data.

## Introduction

Learning textual representations is a vital part of natural language processing (NLP) and important for subsequent NLP tasks. Recently, the study of representations of phrases and sentences has attracted the attention of many researchers, who have achieved a degree of success [1].

Studies of short textual representations have attained a number of achievements, and Miklov’s continuous bag of words (CBOW) model and the skip-gram model (continuous skip-gram model) are among the most famous models. The word representations learned from these models present a relatively good performance in many NLP tasks, including word analogies [2, 3]. Recently, interests have shifted towards extensions of these ideas beyond the individual word-level to larger bodies of text, such as sentences. Researchers hope to directly learn sentence representation via the sum or average based on the word representation, and they have achieved satisfactory results for certain simple NLP tasks [4]. Because of the variable length and complex structure of sentences, these simple algorithms cannot handle complex tasks (such as evaluating the similarity between two sentences). To resolve this problem, Kiros, Tai and Le have proposed methods of learning fixed-length sentence representations [5–7].

Among all models for learning sentence representations, recurrent neural network (RNN) models, especially the Long Short-Term Memory (LSTM) model [8], are among the most appropriate models for processing sentences, and they have achieved substantial success in text categorization [9] and machine translation [10]. Therefore, this paper has also introduced LSTM networks into a dependency-based Siamese LSTM model (D-LSTM) for better performance.

In this paper, a sentence is composed of two parts, namely, the basic component and the supporting component. We have improved upon the traditional method, which employs standard LSTM to learn sentence representations, and proposed the D-LSTM, which is based on sentence dependency to learn sentence representations. The D-LSTM can read sentences with different lengths to generate fixed-length representations. The basic component, which contains fundamental information about a sentence, is obtained by the standard LSTM language model. The supporting component contains the main sentence information (primarily from the subject, predicate and object of a sentence) and is generated after performing a dependency parse on the sentence. While generating the sentence representation, the basic component occupies the dominant position and the supporting component plays a supporting role. This paper has introduced a weight factor (α) that can adjust the ratio of the basic component to the supporting component in the sentence representation to learn the final sentence representation. In the sentence similarity task, the sentence representation learned by D-LSTM has achieved suitable results.

The key contribution of this study is the division of the sentence representation into two parts, i.e., the basic component and the supporting component. Inspired by this idea, this paper proposes the D-LSTM model, which can capture richer information about a sentence than the standard LSTM model and learn an efficient sentence representation. The effects of the different proportions of the basic component and the supporting component in the sentence representation have been carefully investigated via a series of experiments. The addition of a supporting component in the basic representation can improve the performance of the sentence representation.

### Related work

After Bengio proposed the neural probabilistic language model [11], the popularity of neural networks for learning text representations increased. Recently, numerous achievements have been made in learning word-level representations by neural networks, such as the famous CBOW model [3]. Because of the natural advantages of RNNs and LSTMs in sequence processing, researchers have begun to apply RNNs and LSTMs to learn better sentence representations. For example, Kiros proposed the skip-thoughts model, which can extend the skip-gram approach of word2vec from the word level to the sentence level [5]. RNNs adapt standard feedforward neural networks for sequence data (*x*_1_,…*x*_*T*_), and at each t ∈ {1,…,*T*}, updates to a hidden-state vector *h*_*t*_ are performed via

$$h_{t}=sigmoid(Wx_{t}+Uh_{t-1})$$ (1)

Although RNNs present satisfactory performance in handling sequences, they also present a considerable flaw related to long-term dependency, which has been discussed by many researchers [12]. LSTMs networks are explicitly designed to avoid the long-term dependency problem. Similar to RNNs, the LSTM sequentially updates a hidden-state representation; however, these steps also rely on a memory cell that contains four components (which are real-value vectors): the memory state *c*_*t*_ and the output gate *o*_*t*_, which determine how the memory state affects other units, and the input and forget gates *i*_*t*_ and *f*_*t*_, respectively, which control what is stored in and omitted from memory based on each new input and the current state. The following updates were performed at each t ∈ {1,…,*T*} in a LSTM parameterized by the weight matrices *W*_*i*_,*W*_*f*_,*W*_*c*_,*W*_*o*_,*U*_*i*_,*U*_*f*_,*U*_*c*_,*U*_*o*_ and bias vectors *b*_*i*_,*b*_*f*_,*b*_*c*_,*b*_*o*_:

$$i_{t}=sigmoid(W_{i}x_{t}+U_{i}h_{t-1}+b_{i})$$ (2)

$$f_{t}=sigmoid(W_{f}x_{t}+U_{f}h_{t-1}+b_{f})$$ (3)

$$\tilde{c_{t}}=tanh(W_{c}x_{t}+U_{c}h_{t-1}+b_{c})$$ (4)

$$c_{t}=i_{t}ʘ\tilde{c_{t}}+f_{t}ʘc_{t-1}$$ (5)

$$o_{t}=sigmoid(W_{o}x_{t}+U_{o}h_{t-1}+b_{o})$$ (6)

$$h_{t}=o_{t}ʘtanh(c_{t})$$ (7)

Many LSTM variants are available. One popular variant introduced by Gers and Schmidhuber adds “peephole connections” [13]. Another variant is the gated recurrent unit (GRU) [14]. Although many LSTM variants are available, Greff performed a comparison of popular variants and identified their similarities, and the results indicated that the forget gate and output activation function may be the most vital components in LSTMs [15].

Recently, a similar approach to neural network methods has achieved remarkable improvements in performance. Tai, Socher, and Manning (2015) proposed the Tree-LSTMs, which generalize the order-sensitive chain-structure of standard LSTMs to tree-structured network topologies [6]. Each sentence is converted into a parse tree (using a separately trained parser), and the Tree-LSTM composes its hidden state at a given tree node from the corresponding word and the hidden states of all child nodes. Compared with the standard LSTM model, Tree-LSTMs present a forget gate for each child node, which enables the Tree-LSTMs to selectively obtain information about the child’s node and produce a better sentence representation.

With the development of neural networks, a new Siamese Network architecture is also employed for learning sentence representations [16, 17]. Kenter, Borisov and Rijke proposed the Siamese Continuous Bag of Words (Siamese CBOW) model, which was based on the Siamese Network [18]. Their work highlighted that word embeddings trained with the currently available methods are not optimized for the task of sentence representation, whereas Siamese CBOW handles this problem by directly training and then averaging word embeddings. The underlying neural network learns word embeddings by predicting the surrounding sentences from a sentence representation.

**Fig 1:**
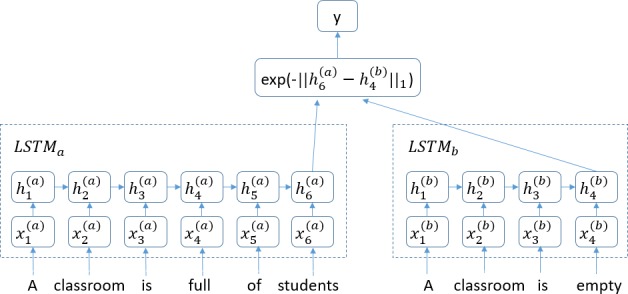
MaLSTM model. The MaLSTM use a LSTM to read in word-vectors that represent each input sentence and employs its final hidden state as a vector representation for each sentence. The similarities among these representations are employed as predictors of semantic similarity.

Jonas and Aditya combined the Siamese Network with LSTMs and proposed their Manhattan LSTM model (MaLSTM) for modelling the semantic similarity among sentences [19]. The MaLSTM model is shown in Fig 1.

Two LSTMs are used in the MaLSTM: *LSTM*_*a*_ and *LSTM*_*b*_ (*LSTM*_*a*_ = *LSTM*_*b*_ in their experiment). Each LSTM processes a sentence in the input sentence pair. The LSTM learns a mapping from the space of variable length sequences of *d*_*in*_-dimensional vectors into $R^{d_{rep}}$ (*d*_*in*_ = 300, *d*_*rep*_ = 50). Each sentence (represented as a sequence of word vectors) *x*_1_,.…,*x*_*T*_ is passed to the LSTM, which updates its hidden state at each sequence-index via Eqs (2)–(7). The final representation of the sentence is encoded by $h_{T}\in R^{d_{rep}}$, which is the last hidden state of the model. For a given pair of sentences, a pre-defined similarity function ${g:R}^{d_{rep}}\times R^{d_{rep}}\to R$ is applied to the LSTM representations (in their study, $g(h_{T_{a}}^{\left(a\right)},h_{T_{b}}^{\left(b\right)})=\exp\left(-{\left\|h_{T_{a}}^{\left(a\right)}-h_{T_{b}}^{\left(b\right)}\right\|}_{1}\right)\in [0,1]$). Similarities in the representation space are subsequently employed to infer the sentences’ underlying semantic similarities. Empirically, the results are fairly stable across various types of simple similarity functions; however, the function g, which utilizes the Manhattan distance, slightly outperforms other reasonable alternatives, such as cosine similarity [20]. In addition, methods of pre-training and synonym expansion for similar datasets have been applied to expand limited training data.

## Materials and methods

### Dependency-based Siamese LSTM model

**Fig 2:**
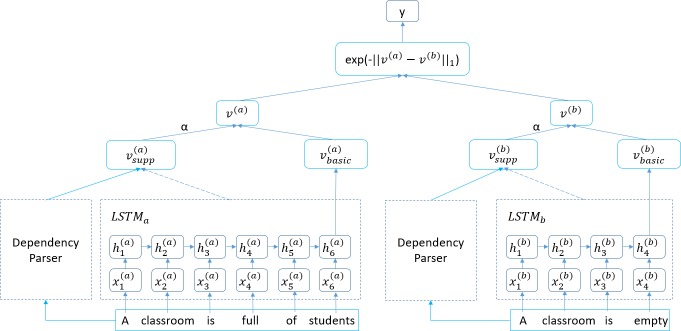
Dependency-based LSTM model. The D-LSTM uses a LSTM to read in word vectors and employs its final hidden state as a basic component ***v_basic_*** for each sentence. The D-LSTM performs a dependency parse on the input sentence and generates the supporting component ***v_supp_*** for each sentence. By introducing the weight factor **α**, the D-LSTM generates the sentence representation ***v*** according to ***v_basic_*** and ***v_supp_***, and it then predicts the similarities among these representations.

The LSTM model has a natural advantage in handling sequences, such as sentences. Compared with words, sentences have more complex structures, and a variety of relations are observed among words in the same sentence. To learn more powerful sentence representations, the difference between sentences and words should be considered. As previously mentioned, a complete sentence representation has two parts: the basic component *v*_*basic*_, which contains the basic sentence information, and the supporting component *v*_*supp*_, which contains the main sentence information (primarily from the subject, predicate and object of a sentence). Based on this idea, this paper proposes the D-LSTM as shown in Fig 2.

A similar network structure as that in Mueller et al. [19] is used to design the D-LSTM model. The D-LSTM reads sentences a and b using two LSTMs and generates fixed-length vectors $v_{basic}^{\left(a\right)}$ and $v_{basic}^{\left(b\right)}$ as the basic components of a and b, respectively (described in another section). While generating the basic component, the D-LSTM also performs a dependency analysis of a and b to obtain the relations among the words in the sentence. The D-LSTM generates the supporting components $v_{supp}^{\left(a\right)}$ and $v_{supp}^{\left(b\right)}$ by summarizing the hidden states that correspond to the input words that have a specific relationship in the sentence (described in another section).

In this study, the basic component of a sentence occupies the dominant position and the supporting component plays a supporting role. Thus, the D-LSTM introduces the weight factor α, which can adjust the ratio of the basic component to the supporting component in the sentence representation to generate the complete sentence representation. The final sentence representation can be calculated using the following formula:

$$v=v_{basic}+\alpha ∙v_{supp}$$ (8)

When *α* = 0, D-LSTM = MaLSTM.

The output layer of the D-LSTM can be changed according to the specific problem. This study investigates sentence similarity, and the similarity between *v*^(*a*)^ and *v*^(*b*)^ can be calculated using the similarity function $Sim(v^{\left(a\right)},v^{\left(b\right)})=e^{-{\left\|v^{\left(a\right)}-v^{\left(b\right)}\right\|}_{1}}$.

The basic component and supporting component of a sentence are described in the following subsections.

### Basic component

The basic component contains basic information about a sentence. A common method of obtaining the basic component is the bag-of-words model, which does not consider the order of the words in the sentence and directly obtains the sentence representation by summarizing (or applying another mathematical calculation) the word representation that corresponds to the word. However, sentence representations obtained in this manner disregard important information, such as the order of each word in the sentence. To solve this problem, the LSTM model is chosen in this study to learn the basic component of a sentence. First, the D-LSTM converts each word in the input sentence into a word embedding (in the experiment, pre-trained word2vec vectors were employed). Second, the D-LSTM updates its memory state *c*_*t*_ and hidden state *h*_*t*_ at each t ∈ {1,2,…,*T*} according to formulas (2)–(7). Finally, the model generates set *C* = {*c*_1_,*c*_2_,…,*c*_*T*_}, which contains all the memory states, and set *H* = {*h*_1_,*h*_2_,…,*h*_*T*_}, which contains all the hidden states, where T represents the total number of words in the sentence.

Because the LSTM receives the inputs in sequence and generates the memory states and hidden states at the current time based on the output at the previous time, the memory states and hidden states that are generated at a particular moment contain all previously entered information. Therefore, this paper chooses *v*_*basic*_ = *h*_*T*_ as the basic component of the sentence, which is similar to that of Mueller et al. [19]. For example, as shown in Fig 2, $v_{basic}^{\left(a\right)}=h_{6}^{\left(a\right)}$ and $v_{basic}^{\left(b\right)}=h_{4}^{\left(b\right)}$.

### Supporting component

In addition to the basic component, the supporting component contains supporting information in the sentence representation in this paper. To learn the supporting component of the sentence, the Stanford Parser [21], which is a natural language parser that determines the grammatical structure of sentences, such as the groups of words that go together (e.g., as “phrases”) and the words that represent the subject or object of a verb, is used to perform a dependency parse on the sentences. A total of 37 universal syntactic relations, such as the nominal subject (nsubj), object (obj) and indirect object (iobj), are observed. From a linguistic perspective, a sentence is composed of different components, such as a subject, predicate, object, attributive adjective, adverbial phrase and complements. Of all the components, the subject, predicate and object serve the most important roles in a sentence; thus, the D-LSTM labels the words in the analysis that have the relation *subj (including nominal and clausal subject), which can identify the subject of a sentence, and *obj (including direct object and indirect object), which can identify the predicate and object of a sentence and generates the one-hot vector *v*_*d*_. The D-LSTM generates the supporting component by the following formula:

$$v_{supp}=\frac{1}{\sum v_{d_{i}}}\sum_{i=1}^{T}v_{d_{i}}∙h_{i}$$ (9)

where $v_{d_{i}}\in \{0,1\}$ is the value at the i-dimensional dimension of the *v*_*d*_ and *h*_*i*_ ∈ *H* is the hidden state that corresponds to the i-th word. The results produced by the Stanford Parser are listed in Table 1.

**Table 1:** Results produced by the Stanford Parser.

A classroom is full of students	A classroom is empty
(('full', 'JJ'), 'nsubj', ('classroom', 'NN'))	(('empty', 'JJ'), 'nsubj', ('classroom', 'NN'))
(('classroom', 'NN'), 'det', ('A', 'DT'))	(('classroom', 'NN'), 'det', ('A', 'DT'))
(('full', 'JJ'), 'cop', ('is', 'VBZ'))	(('empty', 'JJ'), 'cop', ('is', 'VBZ'))
(('full', 'JJ'), 'nmod', ('students', 'NNS'))	-
(('students', 'NNS'), 'case', ('of', 'IN'))	-

Each triplet in the table represents a dependency that occurs in the sentence. For example, in the triplet (('full','JJ'), 'nsubj', ('classroom', 'NN')), ‘JJ’ and ‘NN’ denote the parts of speech of the corresponding word and ‘nsubj’ denotes the relation between ‘full’ and ‘classroom’.

According to the analysis results shown in Table 1, $v_{d}^{\left(a\right)}=(0,1,0,1,0,0)$ and $v_{d}^{\left(b\right)}=(0,1,0,1)$. The supporting components can be calculated according to formula (9):

$$\begin{array}{c} v_{supp}^{\left(a\right)}=\frac{1}{0+1+0+1+0+0}v_{d}^{\left(a\right)}∙{\left(h_{1}^{\left(a\right)},h_{2}^{\left(a\right)},h_{3}^{\left(a\right)},h_{4}^{\left(a\right)},h_{5}^{\left(a\right)},h_{6}^{\left(a\right)}\right)}^{T}=\frac{1}{2}(h_{2}^{\left(a\right)}+h_{4}^{\left(a\right)})\\ v_{supp}^{\left(b\right)}=\frac{1}{0+1+0+1}v_{d}^{\left(b\right)}∙{\left(h_{1}^{\left(b\right)},h_{2}^{\left(b\right)},h_{3}^{\left(b\right)},h_{4}^{\left(b\right)}\right)}^{T}=\frac{1}{2}(h_{2}^{\left(b\right)}+h_{4}^{\left(b\right)}) \end{array}$$

### Experiment

#### Data

Two data sets are employed in the experiment: a sentence involving a compositional knowledge (SICK) data set and a pre-training data set.

The SICK data set is a labelled data set that contains 9927 (5000 for training/4927 for testing) pairs of sentences [1]. Each sentence pair is annotated with a relatedness label ∈ [1,5] that corresponds to the average relatedness judged by ten different individuals, and each of the SICK sentence pairs has also been labelled as one of three classes: entailment, contradiction, or neutral, which are to be predicted for the test examples.

The pre-training data set consists of separate sentence-pair data provided for the previous SemEval 2013 Semantic Textual Similarity task. The pre-training data set contains approximately 11000 pairs of sentences that also have a label ∈ [1,5] [22].

### Semantic relatedness scoring

#### Evaluation metrics

There are three evaluation metrics in the semantic relatedness task: the Pearson correlation coefficient, the Spearman correlation and the mean squared error (MSE). The Pearson correlation coefficient is the official ranking basis and we mainly evaluate the model based on the Pearson correlation coefficient.

The Pearson correlation coefficient (PCC), which is also referred to as the Pearson’s r, is a common metric for the semantic textual similarity tasks, and the Pearson product-moment correlation coefficient (PPMCC), or the bivariate correlation, is a measure of the linear correlation between the two variables X and Y. The PPMCC has a value between +1 and -1, where 1 represents a total positive linear correlation, 0 denotes no linear correlation, and -1 represents a total negative linear correlation [23]. The goal of the task is to obtain the largest possible PCC for the test set.

The Spearman rank correlation coefficient between two variables is equal to the Pearson correlation between the rank values of those two variables [24]; whereas the Pearson correlation assesses linear relationships, the Spearman correlation assesses monotonic relationships (whether linear or not). If there are no repeated data values, a perfect Spearman correlation of +1 or −1 occurs when each of the variables is a perfect monotonic function of the other. Intuitively, the Spearman correlation between two variables will be high when observations have a similar (or identical, for a correlation of 1) rank (i.e., relative position label of the observations within the variable: 1st, 2nd, 3rd, etc.) between the two variables, and low when observations have a dissimilar (or fully opposite, for a correlation of −1) rank between the two variables.

The MSE of an estimator (of a procedure for estimating an unobserved quantity) measures the average of the squares of the errors—that is, the difference between the estimator and what is estimated. MSE is a risk function, corresponding to the expected value of the squared error loss or quadratic loss [25].

#### Training details

The D-LSTM has two versions: the D-LSTM with pre-trained data and the D-LSTM without pre-trained data.

The parameters of the D-LSTM are initialized with a Gaussian distribution (μ = 0.0, σ = 0.02) and a separate large value of 2.5 for the forget gate bias to facilitate the modelling of long-range dependence. In the pre-training version, the pre-training data set is used to pre-train the model, and the pre-trained model will continue to be trained as the initial model of the training phase.

In the training phase, the 300-dimensional word2vec embeddings are employed, and they are not updated during the training process. The D-LSTM uses the 50-dimensional hidden representations *h*_*t*_ and the memory cells *c*_*t*_. The parameter optimization is performed using the Adadelta method of Zeiler [26] and gradient clipping (rescaling gradients in which the norm exceeds a threshold) to avoid the exploding gradients problem [27].

#### Results

We implement the MaLSTM (without regression calibration and synonym augmentation) and D-LSTM (pre-trained and no pre-trained versions) with Tensorflow. The code will soon be made publicly available.

Pearson’s r values, the Spearman correlations and the MSEs for all models using the SICK test data are listed in Table 2. The bolded model names represent models with pre-training, and the numbers in brackets represent the alpha values for the model. The first four models are the top SemEval 2014 submissions [28].

**Table 2:** Pearson’s r values, Spearman correlation and MSE for all models.

Model	PEARSON	SPEARMAN	MSE
ECNU_run1	0.8280	0.7689	0.3250
StanfordNLP_run5	0.8272	0.7559	0.3230
The_Meaning_Factory_run1	0.8268	0.7722	0.3224
UNAL-NLP_run1	0.8043	0.7458	0.3593
**MaLSTM**	0.8211	0.7671	0.3601
**D-LSTM(0.1)**	0.8232	0.7659	0.3569
**D-LSTM(0.2)**	0.8268	0.7678	0.3513
**D-LSTM(0.3)**	0.8280	0.7721	0.3493
**D-LSTM(0.4)**	0.8280	0.7698	0.3488
**D-LSTM(0.5)**	**0.8305**	**0.7729**	0.3442
**D-LSTM(0.6)**	0.8298	0.7693	0.3454
**D-LSTM(0.7)**	0.8292	0.7711	0.3468
**D-LSTM(0.8)**	0.8284	0.7689	0.3479
**D-LSTM(0.9)**	0.8259	0.7663	0.3528
**D-LSTM(1.0)**	0.8217	0.7620	0.3602
MaLSTM	0.8177	0.7585	0.3693
D-LSTM(0.1)	0.8184	0.7583	0.3667
D-LSTM(0.2)	0.8230	0.7659	0.3600
D-LSTM(0.3)	0.8222	0.7637	0.3606
D-LSTM(0.4)	0.8270	0.7673	0.3527
D-LSTM(0.5)	0.8212	0.7599	0.3615
D-LSTM(0.6)	0.8200	0.7580	0.3623
D-LSTM(0.7)	0.8247	0.7638	0.3570
D-LSTM(0.8)	0.8231	0.7631	0.3592
D-LSTM(0.9)	0.8204	0.7579	0.3626
D-LSTM(1.0)	0.8216	0.7593	0.3619

From the results shown in Table 2, **D-LSTM(0.5)** has a better Pearson correlation coefficient and Spearman correlation coefficient on the test set than the top SemEval 2014 submissions and MaLSTM, whether pre-trained or not. At the same time, we noticed that **D-LSTM(0.5)** has a slightly worse MSE than the top 1 SemEval 2014 submission (about 0.019 higher). However, we think the MSE is an unstable metric, and we performed an extra experiment to show that the MSE is less stable than the Pearson correlation. With a total of 4927 samples in the test set, we removed the worst 50 model predictions (approximately 1%) and recalculated the Pearson correlation coefficient and the MSE. Upon doing so, we found that the MSE changed by 7%, while the Pearson correlation coefficient only changed by 1%, which shows that, compared with the MSE, the Pearson correlation coefficient is a relatively stable and reliable evaluation metric. This is also the reason why most similarity tasks use Pearson correlation coefficient rather than the MSE as the main evaluation metric.

### Entailment classification

#### Evaluation metrics

Each of the SICK sentence pairs is also labelled as one of three classes: entailment, contradiction, or neutral. The models are evaluated in terms of classification accuracy. The goal of the task is to obtain the highest accuracy for the test set.

#### Training details

We use the best performing model in the similarity experiment to get the sentence representation $h_{T_{a}}^{\left(a\right)},h_{T_{b}}^{\left(b\right)}$, whereupon we compute the simple features (also successfully used by [6]): the element-wise (absolute) differences, $\left|h_{T_{a}}^{\left(a\right)}-h_{T_{b}}^{\left(b\right)}\right|$. Using only these features, we train a radial-basis-kernel SVM using the same method as [19] to classify the entailment labels.

#### Results

The test set accuracy of all the models are shown in Table 3. The first four models are the top SemEval 2014 submissions [28], the last model is a simple SVM model with features learned by D-LSTM, and the others are the more recently proposed methods [29–31].

**Table 3:** Test set accuracy for the SICK semantic entailment classification.

Model	Accuracy
Illinois-LH_run1	84.6
ECNU_run1	83.6
UNAL-NLP_run1	83.1
SemantiKLUE_run1	82.3
Reasoning-based n-best	80.4
LangPro Hybrid-800	81.4
SNLI-transfer 3-class LSTM	80.8
SVM with MaLSTM features	84.2
SVM with D-LSTM features	**85.2**

From the results of the entailment classification, the simple SVM model with features learned by D-LSTM achieved higher classification accuracy than the other methods. Therefore, the addition of a supporting component does play a role in optimizing sentence representation, and our model can learn more meaningful sentence representations.

## Discussion

### Impact of the supporting component *v*_*supp*_ on the model

**Fig 3:**
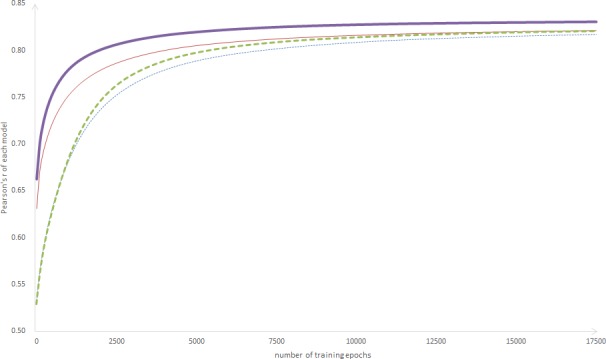
Change of Pearson’s r over training steps. The horizontal axis represents the number of training epochs, and the vertical axis represents Pearson’s r. The solid line indicates the model with pre-training, and the dashed line indicates the model without pre-training (the thick line indicates the D-LSTM, and the thin line indicates the MaLSTM).

To study the changes in model performance after adding the supporting component, this section compares the D-LSTM (α = 0.5) with the MaLSTM. In this experiment, both versions of each model (pre-trained and non-pre-trained) have been investigated, and the results are shown in Fig 3.

The curve indicates that both the performance of the MaLSTM and D-LSTM models can be enhanced by pre-training and shows that D-LSTM has higher training efficiency (Pearson’s r of the D-LSTM is higher than that of the MaLSTM with the same training epochs). When training is finished, the D-LSTM has a higher Pearson’s r than the MaLSTM. Although the standard LSTM does focus sufficient attention on the main structure of the sentence, our D-LSTM incorporates this structure information when generating the sentence representation; thus, a better representation can be obtained.

### Influence of the weight factor α on model training

**Fig 4:**
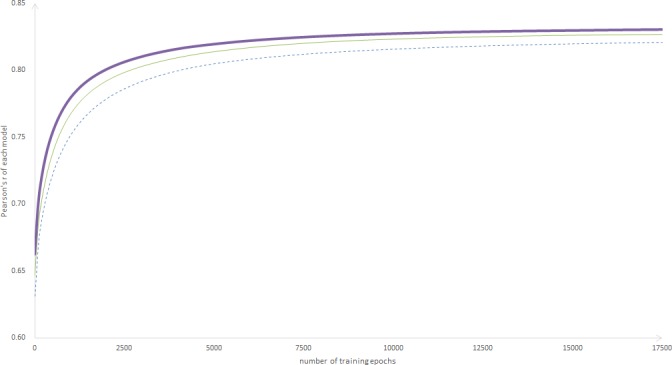
Pearson’s r of different weight factors over training steps. The horizontal axis represents the number of training epochs, and the vertical axis represents the Pearson’s r value. The dotted line indicates the MaLSTM, the thick solid line indicates the D-LSTM with α = 0.2, and the fine solid line indicates the D-LSTM with α = 0.5.

To describe the effects of different weight factors on model training, we selected weight factors of α = 0.0 (the same as in the MaLSTM), α = 0.2, α = 0.5 for further analysis, and the results are shown in Fig 4 (some weight factors are not shown as the curves are partially overlapped).

When the weight factor increases within a certain range, the slope of the curve will increase, which indicates that the parameters of the model have been better optimized. This result demonstrates that the structure information of the sentence (which primarily refers to the subject, predicate and object) has an important impact on the sentence similarity task. With an increase in the proportion of the structure information in the sentence representation, the model can capture more powerful sentence representations to accurately evaluate the similarities among sentences.

### Influence of the weight factor α on model performance

**Fig 5:**
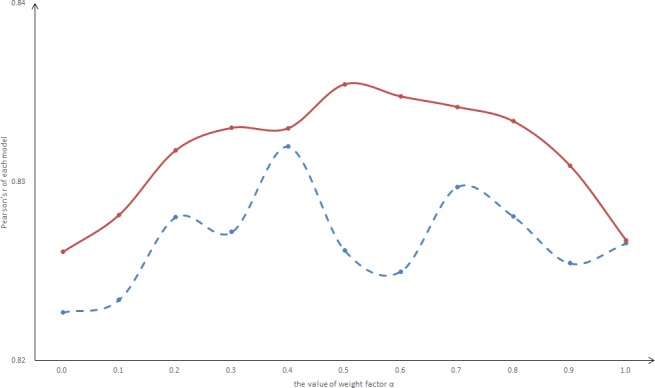
Pearson’s r of D-LSTM with each weight factor. The horizontal axis represents the weight factor, and the vertical axis represents the Pearson’s r. The dotted line indicates the model without pre-training, and the solid line indicates the model with pre-training.

This section chooses α ∈ {0.0,0.1,0.2,…, 1.0} to study the influence of the weight factor on model performance. The Pearson’s r values of the D-LSTM with each α are shown in Fig 5.

As shown in Fig 5, the Pearson’s r values of the D-LSTM in the test set are higher than the Pearson’s r values of the MaLSTM (α = 0.0), regardless of whether the model was pre-trained. Based on the trend of the solid line, the Pearson’s r values of the D-LSTM increase and then decrease. When α ∈ [0.5,0.6], the Pearson’s r value has reached a maximum for the test set. Conversely, the dotted line does not follow a law similar to the solid line. The difference between the solid and dotted lines is that only 9927 pairs of sentences are in the SICK data set without pre-training data, and this number is insufficient for training a suitable LSTM model. Additional pre-training data enhance the ability of the D-LSTM to capture powerful representation.

### Examples of MaLSTM and D-LSTM predictions

To study the specific predictions of D-LSTM and MaLSTM after adding the supporting component, this section selects specific pairs of sentences from the test set as shown in Table 4.

**Table 4:** Examples of MaLSTM and D-LSTM predictions.

Sentence pair	G	M	D
A brown and white dog is running through the water	3.1	3.47	3.14
A dog is emerging from a lake			
A man is lazing	2.8	3.08	2.78
A man is doing exercises			
A small child is showing excitement on a swing set at the park	3.8	3.97	3.81
A small child is showing boredom on a swing set at the park			
A man is cutting a paper plate	3.6	3.69	3.66
The man is not cutting a paper plate			

G denotes the ground truth relatedness ∈ [**1, 5**], M = MaLSTM predictions, and D = D-LSTM (α = 0.5) predictions.

For the first pair, the MaLSTM only focuses on the basic information of the sentence and pays minimal attention to the main components of the sentence. Thus, the adjective “brown and white” before subject “dog” interferes with the MaLSTM predictions, whereas the D-LSTM avoids this problem by paying adequate attention to the subject, predicate and object of the sentence. In the third pair, these two sentences are longer than the first pair, which indicates more noise, such as “small”, in the sentence. However, the D-LSTM exhibits satisfactory performance.

For the second pair, the meanings of the two sentences are opposite but the structure of the two sentences are similar, and although this information is appropriately captured by D-LSTM, the MaLSTM does not recognize this finding. As a result, the D-LSTM yields more accurate predictions than the MaLSTM. The fourth pair of sentences also presents opposite meanings, although the main difference is that the latter has the word “not”, which may have a strong effect on the meaning of the sentence (especially in the sentiment analysis). This difference does affect the MaLSTM predictions. However, the D-LSTM can weaken the effect of “not” on the sentence representation via the dependency analysis, which allows the D-LSTM to focus greater attention on the man/cutting/plate in the sentence than the other components. Thus, the D-LSTM prediction is similar to the true label.

## Conclusions

This paper proposes the novel D-LSTM model for learning powerful sentence representations, which are divided into two parts: a basic component and a supporting component. The D-LSTM learns the basic component and the supporting component of the sentence via different methods. To learn the basic component, the D-LSTM employs the standard LSTM network. To overcome the lack of labelled data, the training data were expanded with additional sentence pairs. To learn the supporting component, the D-LSTM employs a pre-trained Parser to analyse the input sentence, and then it labels the subject, predicate and object in the sentence to generate the dependency representation and finally learns the supporting component. The weight factor α is introduced to adjust the importance of the basic component and the supporting component and learn the sentence representation.

This study experimentally demonstrated that increasing the proportion of the supporting component in the sentence representation increases the power of the representation. The effect of the weight factor α on the training process and results was carefully investigated. The results indicate that increasing the value of the weight factor improves the training efficiency within a certain range as well as the performance of the model. To explain why the performance of the D-LSTM is superior to the standard LSTM, this paper selected pairs of sentences in the test set and compared their predictions. In sentences with more adjectives or turning words, such as “not”, the D-LSTM can weaken the noise and learn more powerful sentence representations, which is useful for identifying the similarities among sentences.
